# A UWB/INS Trajectory Tracking System Application in a Cycling Safety Study

**DOI:** 10.3390/s23073629

**Published:** 2023-03-31

**Authors:** Sicong Zhu, Hao Yue, Tatsuto Suzuki, Inhi Kim, Lei Yu, Qing Lan

**Affiliations:** 1 Key Laboratory of Transport Industry of Big Data Application Technologies for Comprehensive Transport, Ministry of Transport, Beijing Jiaotong University, Haidian District, Beijing 100044, China; hyue@bjtu.edu.cn; 2 Hebei Higher Institute of Transportation Infrastructure Research, Development Center for Digital and Intelligent Technology Application, Cangzhou 061001, China; qinglan@hbwe.edu.cn; 3 Department of Civil, Environ &Geomatic Engineering, Faculty of Engineering Science, London’s Global University, London WC1E 6BT, UK; t.suzuki@ucl.ac.uk; 4 Cho Chun Shik Graduate School of Mobility, Korea Advanced Institute of Science & Technology (KAIST), Daejeon 34141, Republic of Korea; inhi.kim@kaist.ac.kr; 5 Shandong Jiaotong University, No. 5001 Haitang Road, Changqing District, Jinan 250357, China; yulei@sdjtu.edu.cn; 6 College of Transportation Engineering, Hebei University of Water Resources and Electric Engineering, No. 1 Chongqing Road, Cangzhou 061001, China

**Keywords:** UWB/INS, adaptive Kalman filter, accident prevention, chicane

## Abstract

This paper focuses on the safety issue for cyclists and pedestrians at unsignalized intersections. The cycling speed needs to be calmed when approaching the intersection. This study proposes and deploys an integrated portable ultra-wideband/inertial navigation system (UWB/INS) to extract cycling trajectories for a cycling safety study. The system is based on open-source hardware and delivers an open-source code for an adaptive Kalman filter to enhance positioning precision for data quality assurance at an outdoor experimental site. The results demonstrate that the system can deliver reliable trajectories for low-mobility objects. To mitigate accident risk and severity, varied cycling speed calming measures are tested at an experimental site. Based on the trajectory data, the statistical features of cycling velocities are evaluated and compared. A new proposed geometric design is found to be most effective when compared with conventional traffic signs.

## 1. Introduction

Cycling has been revitalized worldwide as a preferred active traffic mode following the large-scale prevalence of the COVID-19 pandemic [1]. As a human-powered and emission-free mode, bicycles are being recommended in more and more countries [2]. The production of bicycles continues to grow significantly from major manufacturing enterprises in China, increasing 19.7% during the first six months of 2021 [3]. The portion of Internet rental bike and public bike ridership has seen a rise of 35.2% compared to the previous year, reaching 730 million trips in 2020 [4].

With the growing level of ridership, infrastructural interventions to bicycle lanes and local crossings are accordingly being redeveloped and expanded [5]. Cyclists face the problem of low stability and are particularly vulnerable when traveling at a lower speed, due to the difficulties of being detected by drivers, while riding at a higher speed may increase the severity of head and neck injuries. Most severe injuries in bicycle accidents involve the head and neck, sometimes leading to fatalities [6]. In most circumstances, cyclists are exposed to great danger when they are involved in accidents. Compared to pedestrians, cyclists more often directly mix with other transportation modes. Cyclists traveling through much more complex mixed traffic increases the risk of cyclist collision and subsequent cyclist falling.

Therefore, this study focuses on two issues. Firstly, a proper solution methodology is proposed to collect trajectory data of low-speed active mode traffic objects, e.g., cyclists and pedestrians. Compared with sophisticated three-dimensional simulators and the widely-used instrumented vehicle in the field, the trajectory collection devices for active mode traffic are relatively underdeveloped. Onboard and remote sensing devices are reviewed for varied experimental scenarios. Secondly, calming approaching speed is essential to improve safety for cyclists and pedestrians. Additionally, permanent traffic facilities that enhance the awareness of speed reduction and safety conditions at unsignalized crossings deserve more in-depth research and attention.

## 2. Literature Review

Conflicts between cyclists and pedestrians can occur at the zebra crossing or the mixed lane [7]. Given the complexity of pedestrian–cyclist interactions in shared space, the research object is the level perpendicular pedestrian crossing because it is common and has high relative speed. The damage to cyclists due to collisions was subject to falling height and crash angle. The evaluation of collision risk can be measured by following indicators, namely, Time-To-Collision (TTC), Post Encroachment Time (PET), Deceleration Rate to Avoid Collision (DRAC), etc.; the collision severity is evaluated by relative speed or kinetic energy. Thus, speed calming is critical for widespread unsignalized intersections where road users are not necessarily adhering to traffic rules. Therefore, current speed-calming methods and cycling trajectory capture techniques are essential for the outdoor scenario.

### 2.1. Road Signs

Accident statistics show that intersections with insufficient sight distance and poor road maintenance [8] increase the risk of conflict. Static road signs, including markings on cycle lanes and crosswalks, and signs established on both sides of the road, regulate cyclist behavior, especially at intersections without traffic signals. Infrastructure at intersections, such as exclusive bicycle lines, crossing signs, and intersection crossing markings, can improve both perceived and actual cycling safety [9]. As discovered by Bian et al. [10], newly designed crosswalk markings in China are comprehensively effective in influencing driver behavior, compared with standard markings. Some current guided signs in rural areas are also regarded as insufficient [11]. Even though roadside signs and road markings are developed to regulate cyclist behavior psychologically, the neglect to impose punishments when signs are not followed is a common phenomenon. The effectiveness of road signs is worth further research, especially on local roads without the control of traffic signals and police.

### 2.2. Road Geometry

The geometric design of lanes, including planetary geometric design and slope types, is also widely studied. Planetary geometric design, also named Chicane, focuses more on lane width and curves, with speed commonly used as a measure to detect the effectiveness of new designs [12,13]. Other studies focus on vertical changes or on raised crossings that reduce speed. However, this type of speed calming may destabilize cycling, and this study excludes it due to the below-mentioned elevation data issue. In terms of cross-sectional design, as discussed by Garcia et al., the minimum lane width should be set at no less than 1.6 m [14] to guarantee that cyclists can ride on their own side on two-way cycle tracks. According to Nolan, et al. [15], the preferable width of a bicycle lane should be no less than 2.3 m, including a wide painted buffer to reduce accidents from cars entering the lane effectively. The protected bike lane adds distance to travel lanes, another special road design that improves safety. Separated bicycle paths are found beneficial in reducing severe crashes as an alternative to cyclists sharing the road with drivers [16]. Madsen and Lahrmann [17] separately evaluated five geometric layouts of bicycle infrastructure with different lengths and widths, concluding that the full-length bicycle track recessed from the parallel road appears to be safer in terms of risk intensity at intersections. However, most studies focus more on safety issues related to motor vehicles, as the trajectories of individual cyclists are hard to trace. Conflicts between pedestrians and cyclists are comparatively overlooked. A limited number of studies focus on geometric design by considering the influence of the lane width, compounded with curve deflections, on cycling speed in the absence of signalized control.

### 2.3. Cyclists Data Collection 

Data quality is critical to trace the trajectories of bicycles. There are three major experiment sites, namely, indoor laboratory, outdoor experimental field, and traffic field, classified by the degree of environmental factors control. PEARL and its successor PAMELA delivered a full control of nature and anthropologic factors for varied traffic scenarios [18]. The outdoor experimental sites establish the traffic facilities to simulate the field. In-situ experiment is also applied to collect trajectories and biometric data in varied studies [19]. A survey demonstrates the state of the art of none –intrusive cyclist speed measuring techniques [20]. Several studies capture cyclists’ behavior using video cameras that record at fixed locations. However, targets are easily obscured with unreliable recognition in video images owing to the variation in subjects’ appearance and the complexity of traffic conditions at intersections [21]. Due to the weather, illumination, and/or shadow, the rapidly changing environment can also cause inaccuracy [22]. Besides technical issues, privacy concerns were raised due to the collection of personal facial data. Thus, this technique has difficult to use in a laboratory for large-scale experiments or at public traffic facilities. Secondly, to capture cyclists’ trajectories, some studies have used portable global positioning system (GPS) sets as a preferred choice. This is usually characterized by a large data sample size, but has relatively low accuracy. So it is suitable for macro-scale research on riding behavior and traffic flow rather than lane-by-lane accuracy.

Considering the above points, studies focusing more on specific individuals tend to use instrumented bicycles to collect continuous data. Two groups of devices are used to capture trajectories. Firstly, LiDAR (light detection and ranging) has been commonly used to track accurate position, speed, and direction. Lee, et al. [23] used stationary LiDAR sensors that help the instrumented bicycle to record cycling maneuvers. However, LiDAR captures geometric information by scanning the environment, resulting in low accuracy in geometrically degenerated cases [24]. Also, LiDAR sensors incur high costs and have high computational needs. 

Trajectory capture devices that are affordable, precise, robust, and portable need to be attached with other devices to instrumented bicycles to study cyclists’ behavior. Zhu and Zhu [25] used an instrumented bicycle equipped with a video camera and sensors, including a GPS receiver, accelerometer, gyro sensor, etc., to collect data to study the influence of the comfort level of cycling infrastructure on cyclists’ perceptions and route choices. To collect data, Lee et al. [23] used an instrumented bicycle with an attached inertial measurement unit (IMU), a potentiometer, and a direct current (DC) motor to study the braking and steering maneuvers performed by cyclists when avoiding obstacles. Different devices can be set and grouped on the bicycle for higher-precision data collection, with the combination of devices selected by researchers continuing to change and improve.

This study focuses on safety improvements for bicycles and pedestrians at unsignalized intersections. As collisions between cyclists and pedestrians can inflict mutual damages, appropriate speed calming is critical for cycling safety. Among varied factors, the study focuses on quantifying the speed calming methods that affect and reduce the final speed of bicycles immediately at the conflicting crossing for unsignalized intersections. The infrastructure speed calming study comprises static deceleration-warning signs, light-emitting diode (LED)-enhanced active warning signs, and, in particular, a new geometric design for the bicycle lane. The study also proposes a comprehensive solution for varied experimental scenarios for this long-time unremarkable research object. The current trajectory tracking video and Lidar can reach cm-level precision [24,26]. The study requires ultra-wideband (UWB) modules with inertial measurement units (IMUs) to capture the trajectories of bicycles and other active mode traffic with high precision and robustness in outdoor experimental scenarios. This study conducts statistical analysis on the cycling speed at unsignalized intersections after the trajectory data are filtered for quality assurance.

## 3. Experimental Setup

### 3.1. Participants

For each road infrastructure scenario, approximately 30 participants were recruited from the Beijing Jiaotong University (BJTU) campus. Each participant was asked to ride the instrumented bicycle while performing braking or steering avoidance maneuvers at two different speeds. Selection criteria required young participants to be between the ages of 20 and 35 years to exclude the effect of age. They needed to be regular cyclists and be able to ride the instrumented bicycle with caliper brakes comfortably. In the field, the data were collected from approximately 30 participants from the BJTU campus for each scenario. The participants comprised 64% males and 36% females. The ages of the qualified participants ranged from 18 to 24 years, and all were reported to be frequent cyclists.

### 3.2. Devices and Data Collection

**Figure 1:**
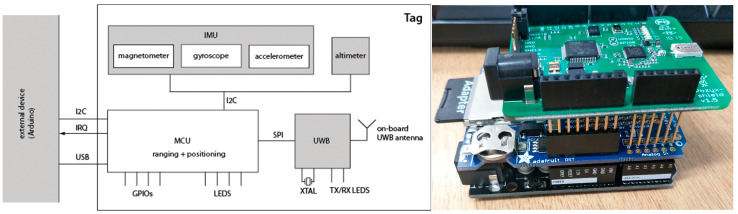
The architecture of data logging system and device, adapted with permission from [28].

A data logging platform collected data via the UWB tag mounted on an instrumented bicycle. The UWB tag and logging shield ran a C code on an Arduino microcontroller (MCU) [27], shown in Figure 1.

**Figure 2:**
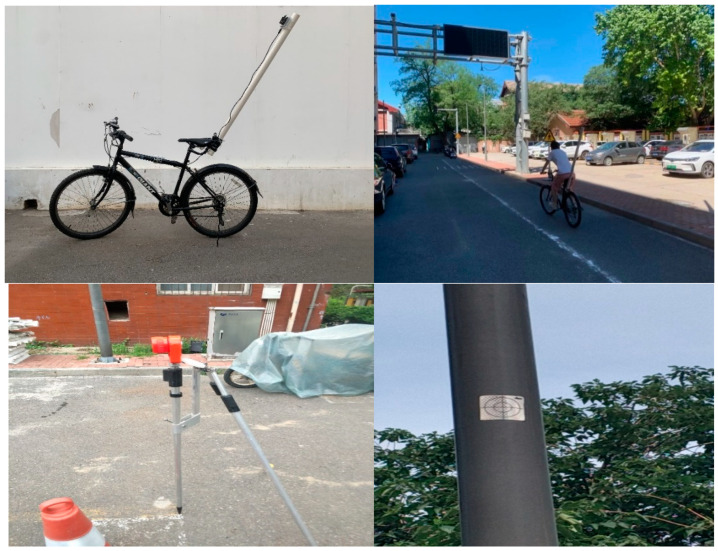
Instrumented bicycle and experimental site setup.

Data were collected from the UWB tags, and a push button could reset the system for synchronized logging. A UWB component, including an antenna and a signal processing unit, was mounted to calculate the positioning information. An IMU was also on the board setting to measure the bicycle’s motions. The IMU measured acceleration within a range of ±4 g, with the angular rate within a range of ±2000 deg/s. The update rate of the accelerometer and gyroscope sensors can reach 100 Hz [28]. The instrumented bicycle was an urban 18-speed bicycle with both front and rear brakes, as shown in Figure 2. The UWB tag was mounted to the instrumented bicycle rigidly. The upwards plastic bar is electromagnetic immunity and provides a clear line of sight. The UWB anchors broadcast signals, and their positions were set out using mm-precision total station and mounted to poles for a clear line of sight to the roaming tags, shown in Figure 2.

### 3.3. Experimental Protocol

During the experiment, the participants were asked to ride the instrumented bicycle on a straight path before entering the experimental area as a get-ready and accelerating section. The section design was deflected to better control and limit the speed. Based on AASHTO bike lane design guidelines, the stopping sight distance can be calculated as 16.3 m for a cyclist traveling at 15 km/h [29]. Thus, a crosswalk of high-quality level asphalt pavement was located 20 m ahead for enough room for cyclists to stop. It was connected to the other side of the experimental area as a place for conflict scenarios between cyclists and pedestrians, as shown in Figure 2. The experiments were categorized using varied speed-calming methods. Standard road marking is the baseline. Additionally, three prognosis studies are static signs, LED-enhanced active warning signs, and, in particular, a new geometric design for the bicycle lane. Both passive and active signs were attached to a pole at the roadside. 

According to the geometry design standards and guidelines, the components of level road geometry consists of straight line, circular curve, and transitional curve. The cycle lane is viewed as an extension of the motor roadway, and the cycle lane’s width is usually lower than the motor vehicle lane [30]. The alignment of the cycle lane is redesigned to use straight lines and circular curves. In particular, the radius of the circular curve can regulate the design speed due to centrifugal force. If the radius is excessively large, the curvy road segment can be shortcut by straight traveling; if the radius is excessively small, the road segment can cause uncomfortable deceleration, even falling from bicycles. Thus, the proposed geometry was drafted by trial and error in the field.

**Figure 3:**
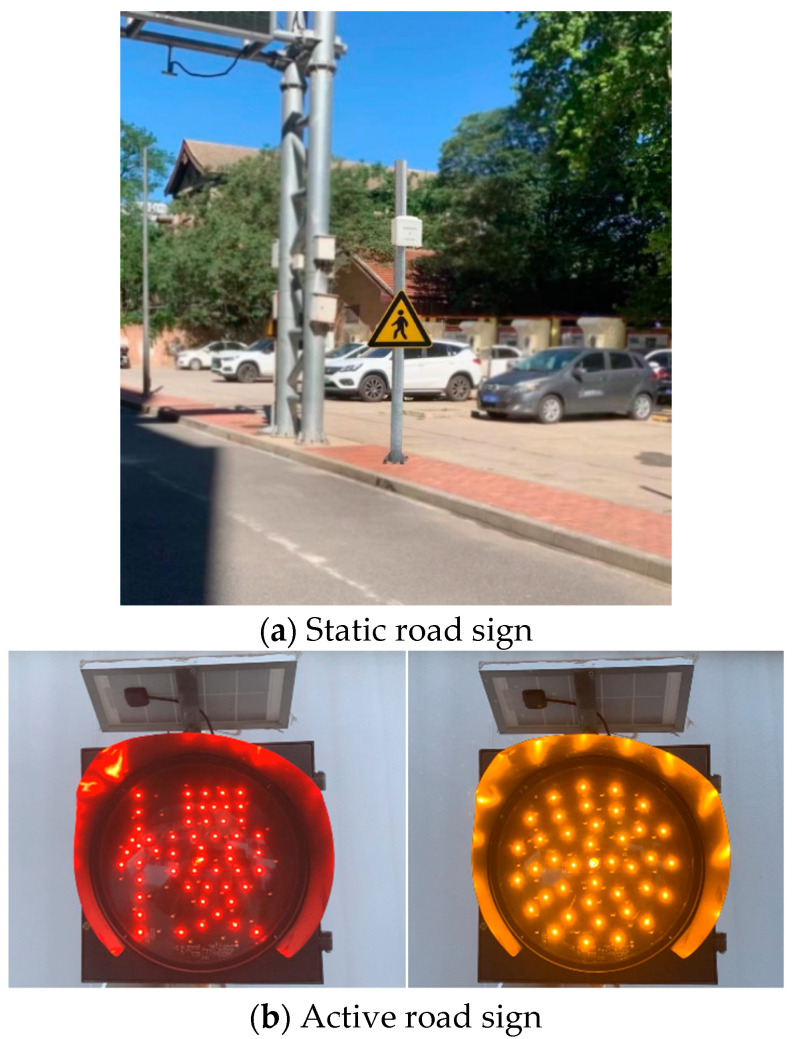

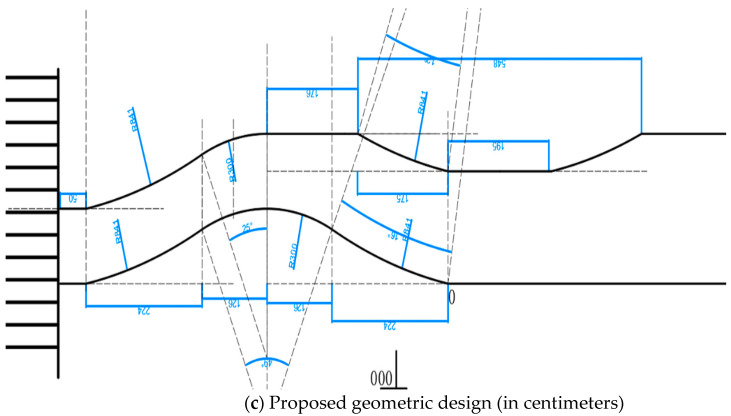
Traffic calming measures.

The entire geometry consists of a curb extension and a serpentine curve. The curb extensions extend the pedestrian sidewalk into the cycling lane at an intersection. It forces cyclists to travel on the left side of the cycling lane. The serpentine-curve chicane separated from the motorway requires the cyclist to turn slightly right and then slightly left to continue on the road (for right-hand traffic). The proposed geometry consists of three varied sub-circular curves, starting with a 5.48 m long curb extension to narrow cycling lane and a 3 m radius curve of 49 degrees to the intersection, considered to be effective for reducing cyclist approaching speed, as presented in Figure 3. The experiments were conducted during the daytime with normal daylight and without road water for normal skid force.

Before the experiment commenced, participants had a session to get used to the dynamics and control of the instrumented bicycle. They practiced braking, steering, and cruising at preset speeds (by monitoring the speedometer attached to the handlebar) until they felt confident to conduct the experiment. Since the mean speed of cyclists in urban areas is approximately 2.8–5.6 m/s [19]. Each participant performed four trials. In the order given by the instructor, the participants cycled at one of the two preset speeds at the experimental site, namely, speed lower than 4.2 m/s (15 km/h) and greater than 4.2 m/s. The participant was instructed to try to keep to the cruising speed for each trial. Participants were asked to cruise, brake, or steer comfortably as they usually do when riding bicycles in traffic. They were also required to come to a full stop before the end of the track and to follow the middle lane of the lane geometry for braking maneuvers. The order of speeds was predetermined from a list of all possible combinations to appear random to participants. Participants were informed about the deployment of traffic management measures when this occurred. Some pedestrians transversed the road at the crossing marked area during the experiment to remind. Some trials were excluded from the analysis due to technical issues or a failure to follow instructions.

## 4. Methodology

### 4.1. Data Processing

The measurement data were processed to obtain safety metrics. The cyclists’ deceleration behavior was quantified by analyzing the speed and deceleration change during braking maneuvers in conflict areas. Before the analysis, the trajectory data were checked to remove errors and enhanced through the adaptive Kalman filter (AFK).

### 4.2. Kalman Filter Description

Ultra-wideband (UWB) uses high-frequency pulses for information transmission but has poor penetration ability and is prone to be significantly influenced by the environment. Non-line-of-sight (NLOS) conditions from blocking by objects between tags and anchors would significantly disturb the positioning results and cause errors. To ensure accuracy of the location data, this study used the simplified Sage–Husa adaptive Kalman filter (SSHAKF) to fuse and compensate for UWB data with the IMU module location results. In NLOS conditions, the IMU ensures the continuity of UWB location results. At the same time, as the acceleration data provided by the IMU need to be accumulated through two rounds of integration to obtain the displacements, errors might diverge rapidly during the integrating process. The IMU errors can also be fused and weakened by UWB through the filter. Besides the NLOS errors, UWB is subject to electromagnetic interference in outdoor environments.

Although the update rate can be 100 Hz in theory, the filter in this study ran at approximately 10 Hz due to the MCU’s limited processing capacity. The state vector estimation used only the state variables in an automatic process. As conventional navigation coordinates, the horizontal and vertical components were the X (North), Y (East), and Z (Up) orientation of the object’s location. As cyclists and pedestrians are usually run at a constant speed on a horizontal plane, only two-dimensional states were considered in this study. The body frame coordination is transformed to UWB system coordination set by total station. The filter’s evolving state is described by the vector in Equation (1)**.**

(1) $$X=(\delta s_{x},\delta v_{x},\delta s_{y},\delta v_{y}{)}^{T}$$

where

$\delta s_{x}$ is the error of position in *X*;

$\delta s_{y}$ is the error of position in *Y*;

$\delta v_{x}$ is the error of speed in *X*;

$\delta v_{y}$ is the error of speed in *Y*.

The prediction and measurement equations are described as:

(2) $$X_{k+1}=AX_{k}+w_{k}$$

(3) $$z_{k}=HX_{k}+v_{k}$$

where

*A* is the transform matrix;

*H* is the measurement matrix;

$w_{k}$ is the process noise at *k* $w_{t}\sim N\left(0,Q\right)$ Q is the variance–covariance matrix

$e_{k}$ is the measurement noise at *k*, $e_{t}\sim N\left(0,R\right)$ R is the variance–covariance matrix

*k* is the number of iteration steps;

$z_{k}$ is the measurement result matrix.

The state transform matrix A and measurement matrix *H* are defined as:

(4) $$A=\left[\begin{array}{cccc} 1 & △t & 0 & 0\\ 0 & 1 & 0 & 0\\ 0 & 0 & 1 & △t\\ 0 & 0 & 0 & 1 \end{array}\right]$$

where

∆*t* is the output interval of the system;

Since the system adopts the UWB coordination, the setup is demonstrated in Figure 2.

(5) $$H=\left[\begin{array}{cccc} 1 & 0 & 0 & 0\\ 0 & 1 & 0 & 0\\ 0 & 0 & 1 & 0\\ 0 & 0 & 0 & 1 \end{array}\right]$$

where ∆t is the output interval of the system, and observation is composed of the difference between UWB and inertial navigation system (INS) output values:

(6) $$z=(s_{x}^{UWB}-s_{x}^{INS},\delta v_{x}^{UWB}-\delta v_{x}^{INS},s_{y}^{UWB}-s_{y}^{INS},\delta v_{y}^{UWB}-\delta v_{y}^{INS})$$

where

$s_{x}^{UWB}\;{and\;s}_{y}^{UWB}$ are the position of UWB output in *X* and *Y*, respectively;

$s_{x}^{IMU}\;{and\;s}_{y}^{IMU}$ are the position of IMU output in *X* and *Y*, respectively.

Assume that the process error of the two-dimensional components of acceleration in the navigation coordinate system is known, being $D(\delta a^{Q})$. The initial process covariance Q can be determined as follows [31]:

(7) $$Q=\left[\begin{array}{cc} Q^{\prime } & 0\\ 0 & Q^{\prime } \end{array}\right]$$

And

(8) $$Q^{\prime }=\left[\begin{array}{cc} 1/4\delta t^{4} & 1/2\delta t^{3}\\ 1/2\delta t^{3} & \delta t^{2} \end{array}\right]D(\delta a^{Q})$$

where

$D(\delta a^{Q})$ is the variance of acceleration in *X* or *Y*;

∆*t* is the output interval of the system.

The measurement covariance *R* is determined depending on the characteristic parameters of the sensor used. The initial observed variances of the two-dimensional components located by UWB, which are assumed to be equal, are defined as $D\left(S_{UWB}\right)$, which can be acquired from the UWB configuration manual or offline positioning error statistics. For the noise corresponding to the velocity component, the UWB velocity is decided by the position differential, with the velocity covariance given by:

(9) $$D\left(v_{UWB}\right)=D\left(\frac{s_{UWB}^{k}-s_{UWB}^{k-1}}{△t}\right)=\frac{2}{△t^{2}}D\left(s_{UWB}\right)$$

where

$D\left(v_{UWB}\right)$ is the variance of UWB speed in X or Y;

$s_{UWB}^{k}$ is the UWB position of time k in X or Y.

As the UWB and INS measurements are independent, the noise covariance *R* is set up as:

(10) $$R=\left[\begin{array}{cc} R_{x}^{\prime } & 0\\ 0 & R_{y}^{\prime } \end{array}\right]$$

where

*R* is the measurement covariance matrix;

(11) $$R=\left[\begin{array}{cc} D\left(s_{UWB}\right)+D\left(s_{INS}\right) & 0\\ 0 & D\left(v_{UWB}\right)+D\left(v_{INS}\right) \end{array}\right]$$

Let us suppose that $D(a_{INS})$ is the acceleration error of the two-dimensional component of the INS output, which can be calculated by the INS configuration. Then, the $D\left(s_{INS}\right)$ and $D\left(v_{INS}\right)$ are set up by

(12) $$D\left(v_{ins}\right)=D\left(v_{uwb}^{k-1}+a_{ins}^{k}∆t\right)=D\left(v_{uwb}\right)+{∆t}^{2}D\left(a_{ins}\right)$$

(13) $$D\left(s_{ins}\right)=D\left(s_{uwb}\right)+{∆t}^{2}D\left(v_{uwb}\right)+\frac{1}{4}{∆t}^{4}D\left(a_{ins}\right)$$

whereas $D(a_{INS})$ and $D\left(s_{UWB}\right)$ can both be set offline according to the sensor configurations.

### 4.3. Simplified Sage–Husa Adaptive Kalman Filter (SSHAKF)

The simplified Sage–Husa adaptive Kalman filter (SSHAKF) is an adaptive filter based on the residual and innovation. It can estimate and adjust the covariance simultaneously according to real-time data. However, the computation load poses a challenge for online filtering. As the process noise has its own relatively high stability, more attention should be paid to variation in measurement noise, that is, using a time-varying covariance *R* instead of a constant one. The modification of *R* is as follows.

(14) $$V_{t}=z_{t}-Hx_{t}$$

(15) $$R_{t}=(1-d_{t})R_{t-1}+d_{t}(V_{t}V_{t}^{T}-HP_{t}^{-}H^{T})$$

(16) $$d_{t}=(1-b)/(1-b^{k})$$

where $V_{t}$ is the residual and b is the fading factor with the range 0<b<1 normally, and the value ranges from 0.95–0.99. $P_{t}^{-}$ is the matrix of the prediction process of Kalman filter. The SSHAKF can enhance the filtering performance in the case of time-varying noise statistical characteristics. As the abnormal UWB observation occurs in the NLOS condition, it is unnecessary to update the measurement covariance step by step. Therefore, a type of measurement discriminator is introduced to update *R*. The filter state is closely related to covariance, with the quality of covariance evaluated by matching the actual residual value to the expected value. The discriminator is based on:

(17) $$V_{t}V_{t}^{T}>\gamma tr(E\left[V_{t}V_{t}^{T}\right])$$

The covariance *R* is updated when the discriminator result is true; tr is the trace of the matrix; γ is the reserve coefficient; and γ≥1 means the actual residual value is greater than the expected one. The expected residual is calculated by:

(18) $$E\left[V_{t}V_{t}^{T}\right]=HP_{t}^{-}H^{T}+R_{t}$$

If γ>1, $E\left[V_{t}V_{t}^{T}\right]$ can be acquired by using a sliding window, and calculating the mean of the values involved, supposing that the width of the window is k, and then:

(19) $$E\left[V_{t}V_{t}^{T}\right]=\frac{1}{k}\sum_{i=1}^{k-1}V_{i}V_{i}^{T}$$

If the discriminator is true, then the $R_{t}$ value is updated; otherwise, $R_{t}$ remains at the previous value.

**Figure 4:**
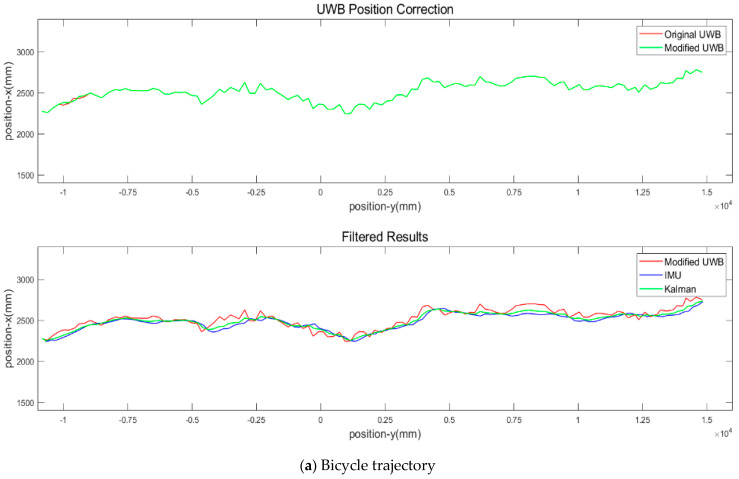

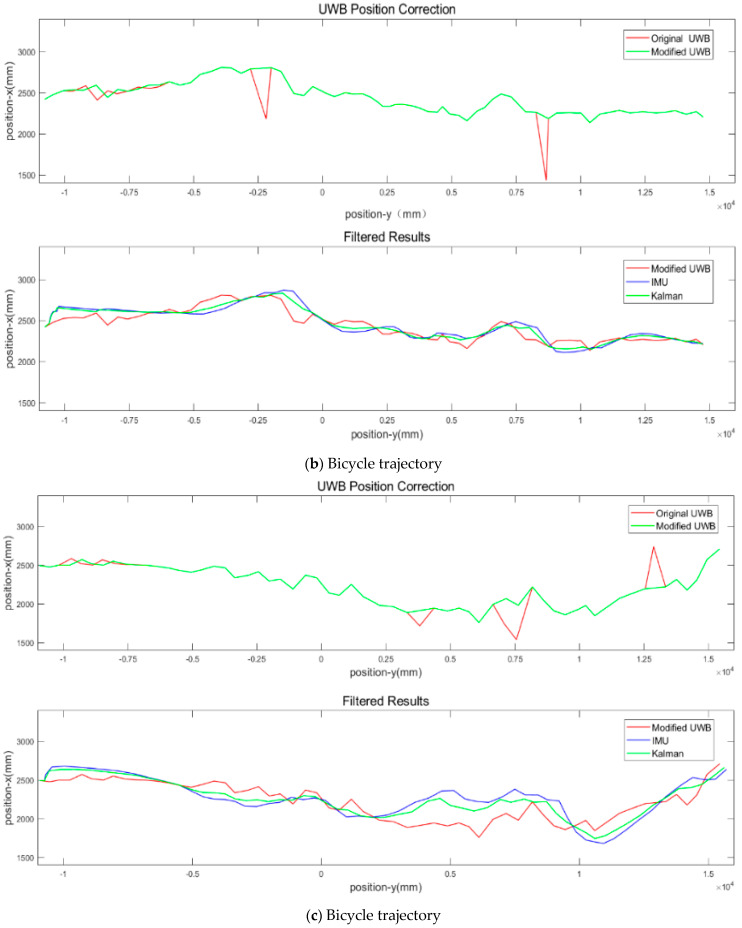
Filtered bicycle trajectories. The algorithm codes are available upon request.

Based on Equation (6), the gap between the UWB measurements and the INS dead reckoning prediction is calculated. If the gap is significantly high, this study assumes that IMU is more reliable due to its electromagnetic wave immunity. The blunders, especially the off-track points, are removed on a quantile basis, with the faulty ones filled by the IMU positioning results. The results are compared with CODA positioning data, and the root mean square error (RMSE) is approximately 20 cm [32]. It is within adult pedestrians’ 50 cm × 60 cm ellipse dimension [33]. Thus, the filtered results can track the entangled trajectories of active mode travelers when yielding and following closely. The SSHAKF is implemented to enhance positioning precision. The smoother field results indicate the more realistic trajectory results for human-powered low-mobility bicycles, presented in Figure 4:

## 5. Experiment Data Analysis and Discussion

The trajectories were collected at the experimental site using varied calming measures. The study focuses on the average speeds at the crossing zone. Therefore, to investigate the significance of the means of the speed in the conflict area, an independent two-sample *t*-test was applied [34]. Specifically, the *t*-test was used to determine whether mean speeds, collected from two experiments, differed from each other at a statistically significant level after the application of the safety measures [35]. The study took the following basic assumptions into account: (a) the samples were unpaired due to participant randomness compounded by data cleaning, and (b) the sample differences could be viewed as a random sample from a population of differences. The results of the f-test and the independent *t*-test are summarized in Table 1 and Table 2.

Null hypothesis: H_0_ = hypothesized value = 0

(20) $$t=\frac{m_{A}-m_{B}}{\sqrt{\frac{S^{2}}{n_{A}}+\frac{S^{2}}{n_{B}}}}$$

(21) $$S^{2}=\frac{\sum {\left(x-m_{A}\right)}^{2}-\sum {\left(x-m_{B}\right)}^{2}}{n_{A}+n_{B}-2}$$

(22) $$df=n_{A}+n_{B}-2$$

where

*A* and *B* = the two groups being compared;

$m_{A}$ and $m_{B}$ = the means of groups A and B, respectively;

$n_{A}$ and $n_{B}$ = the sizes of groups A and B, respectively.

For both the low-speed scenario (a cruising speed less than 4.16 m/s) and the high-speed scenario (a cruising speed greater than 4.16 m/s), two of the three management measures, that is, the passive warning sign and active warning sign, could not reduce the speed significantly in the conflict zone, according to Equation (20). Average speed at crossing was significantly decreased after the deployment of proposed geometry. The risk and severity can be both mitigated. Moreover, the proposed geometry can reduce the variance of speeds significantly compared to the base scenario. It indicates that the proposed chicane can avoid not only excessive high speed but also permit proper low speed that enables bicycles to travel through stably.

Based on these high-resolution cyclist trajectories, the cyclist must usually travel along the left side of the road approaching to curb extension. Moreover, the cyclist on the left side of the road cannot take a shortcut and is forced to enter the varied curvature roadway to reduce speed. The curvature regulates the operation speed not managed by the relationship between centripetal force and speed, as the above-mentioned geometry does not have enough room for the curve radius that fully regulates the approaching speed. Although the radius is not large enough to force cyclists to brake, the cyclists have to change behaviors to keep cruising and stop pedaling, even braking. These improvements indicate that the proposed geometric design delivers effective speed calming for cycling traffic, depending on whether standard operation processes are followed.

## 6. Conclusions

This study discussed cycling speed calming issues and conducted field experiments to verify the effectiveness of road signs and geometric designs based on UWB and IMU modules as tracking devices. After data processing and statistical analysis of reliable trajectory results, several conclusions can be drawn.

The open-source hardware, including its UWB and IMU components, can enhance the precision and robustness of positioning to mitigate electromagnetic interferences in outdoor environments. The system can deliver accurate trajectories to track cyclist trajectories. Moreover, it can deliver a common hardware platform for the research community, which avoids the Galápagos effect, a term of Japanese origin used in business studies to refer to an isolated development branch of a globally available product. The term is an analogy to a finding in Charles Darwin’s *On the Origin of Species* [36]. Unlike the delivery of data by specialized equipment leading to difficulties in repeating experiments to derive results, the common open platform can enable researchers to compare and exchange data with low impedance.

The safety issue for active traffic modes has been largely overlooked as little attention is paid by either the active traffic modes users or by other traffic modes, compared to investigations of the motor vehicle safety issue. On the other hand, the outdoor scenario study of the conflict between active modes faces difficulty in capturing trajectory data during either the trajectory recognition stage or the post-processing stage. This study has proposed a novel application of UWB and IMU modules in studying active mode traffic safety issues and has used the system to track individual trajectories with accuracy and guaranteed continuity. Real-time trajectory data sets acquired by UWB and IMU components can provide research insights on the interaction between pedestrians and cyclists in the conflict zone.

However, the proposed UWB/INS system has been a synchronization issue. The components of the system communicate with the controller unit using different communication protocols. Therefore, the components do not update the outputs simultaneously, but the data collection program reads the registers and records simultaneously. For low-speed active mode, this issue can be neglected in the study, and the portable system should be deployed for validation at intersections to compare the field data with the counterpart data collected at the controlled experimental site. The current study used the aggregated data in terms of average speed. Further study should apply deep data mining for disaggregate dataset. Not only the speed derived from the trajectory but also the bicycle tilt is available for study.

This study’s statistical comparative analysis has shown that static and active road signs barely improve traffic safety by reducing cycling speed. However, the proposed geometric design was effective in reducing cycling approaching speed. Moreover, more consistent approaching speeds indicate that the proposed chicane can regulate speed to reach comfortable cycling at a proper speed. Bicycle lanes with this new geometric chicane could avoid accidents and reduce the severity of accidents through their application at unsignalized intersections and conflict zones. The proposed chicane could be further enhanced through measures such as introducing vegetation and barriers between motor vehicles and cyclists. Before the potential implementation, the capacity loss and installation cost should be taken into consideration.

## Figures and Tables

**Table 1:** Comparison of speed variances at conflict zone.

**High-Speed Scenario**				
**Traffic Management Measure**	**Mean**	**Sample Variance**	***p*-Value**	**Statistical Difference**
				**Alpha 5%**
**Road marking (Baseline)**	4.21 *	0.6	N/A	N/A
**Passive warning sign**	4.30	0.25	0.001	Significant
**Active warning sign**	4.36	0.81	0.168	Insignificant
**Proposed geometric design**	3.27	0.14	6.71 × 10^−11^	Significant
**Low-Speed** **Scenario**				
**Traffic Management Measure**	**Mean**	**Sample Variance**	***p*-Value**	**Statistical Difference**
				**Alpha 5%**
**Road marking (Baseline)**	3.15 *	0.25	N/A	N/A
**Passive warning sign**	3.07	0.39	0.042	Significant
**Active warning sign**	3.03	0.5	0.003	Significant
**Proposed geometric design**	2.78	0.12	0.003	Significant

* Unit m/s.

**Table 2:** Comparison of average speeds at conflict zone.

**High-Speed Scenario**			
**Traffic Management Measure**	**Comparison**	**Statistical Difference (5%)**	***p*-Value**
	**Sample Size**		
**Road marking (Baseline)**	65	N/A	N/A
**Passive warning sign**	56	Insignificant	0.4301
**Active warning sign**	27	Insignificant	0.4142
**Proposed geometric design**	101	Significant	3.55 × 10^−14^
**Low-Speed Scenario**			
**Traffic management measure**	**Comparison**	**Statistical Difference (5%)**	***p*-Value**
	**Sample Size**		
**Road marking (Baseline)**	56	N/A	N/A
**Passive warning sign**	78	Insignificant	0.4198
**Active warning sign**	92	Insignificant	0.2196
**Proposed geometric design**	69	Significant	8.57 × 10^−6^

## Data Availability

The hardware design, algorithm code, and data presented in this study are available on request from the corresponding author. The data are not publicly available due to further publication.
